# A narrative review of research impact assessment models and methods

**DOI:** 10.1186/s12961-015-0003-1

**Published:** 2015-03-18

**Authors:** Andrew J Milat, Adrian E Bauman, Sally Redman

**Affiliations:** New South Wales Ministry of Health, 73 Miller St North, Sydney, NSW 2060 Australia; School of Public Health, University of Sydney, Level 2, Medical Foundation, Building, K25, Sydney, NSW 2006 Australia; Sax Institute, Sydney, Level 2, 10 Quay, St Haymarket, NSW 2000 Australia

**Keywords:** Policy and practice impact, Research impact, Research returns

## Abstract

**Background:**

Research funding agencies continue to grapple with assessing research impact. Theoretical frameworks are useful tools for describing and understanding research impact. The purpose of this narrative literature review was to synthesize evidence that describes processes and conceptual models for assessing policy and practice impacts of public health research.

**Methods:**

The review involved keyword searches of electronic databases, including MEDLINE, CINAHL, PsycINFO, EBM Reviews, and Google Scholar in July/August 2013. Review search terms included ‘research impact’, ‘policy and practice’, ‘intervention research’, ‘translational research’, ‘health promotion’, and ‘public health’. The review included theoretical and opinion pieces, case studies, descriptive studies, frameworks and systematic reviews describing processes, and conceptual models for assessing research impact. The review was conducted in two phases: initially, abstracts were retrieved and assessed against the review criteria followed by the retrieval and assessment of full papers against review criteria.

**Results:**

Thirty one primary studies and one systematic review met the review criteria, with 88% of studies published since 2006. Studies comprised assessments of the impacts of a wide range of health-related research, including basic and biomedical research, clinical trials, health service research, as well as public health research. Six studies had an explicit focus on assessing impacts of health promotion or public health research and one had a specific focus on intervention research impact assessment. A total of 16 different impact assessment models were identified, with the ‘payback model’ the most frequently used conceptual framework. Typically, impacts were assessed across multiple dimensions using mixed methodologies, including publication and citation analysis, interviews with principal investigators, peer assessment, case studies, and document analysis. The vast majority of studies relied on principal investigator interviews and/or peer review to assess impacts, instead of interviewing policymakers and end-users of research.

**Conclusions:**

Research impact assessment is a new field of scientific endeavour and there are a growing number of conceptual frameworks applied to assess the impacts of research.

**Electronic supplementary material:**

The online version of this article (doi:10.1186/s12961-015-0003-1) contains supplementary material, which is available to authorized users.

## Background

There is increasing recognition that health research investment should lead to improvements in policy [1-3], practice, resource allocation, and, ultimately, the health of the community [4,5]. However, research impacts are complex, non-linear, and unpredictable in nature and there is a propensity to ‘count what can be easily measured’, rather than measuring what ‘counts’ in terms of significant, enduring changes [6].

Traditional academic-oriented indices of research productivity, such as number of papers, impact factors of journals, citations, research funding, and esteem measures, are well established and widely used by research granting bodies and academic institutions [7], but they do not always relate well to the ultimate goals of applied health research [6,8,9]. Governments are signaling that research metrics of research quality and productivity are insufficient to determine research value because they say little about the real world benefits of research [10-12]. At the same time, research funders continue to grapple with the fundamental problem of assessing broader impacts of research. This task is made more challenging because there are currently no agreed systematic approaches to measuring broader research impacts, particularly impacts on health policy and practice [13,14].

Recent years have seen the development of a number of frameworks that can assist in better describing and understanding the impact of research. Conceptual frameworks can help organize data collection, analysis, and reporting to promote clarity and consistency in the impact assessments made. In the context of this review, research impact is defined as: *“… any type of output of research activities which can be considered a ‘positive return’ for the scientific community, health systems, patients, and the society in general*” [13], p. 2.

In light of these gaps in the literature, the purpose of this narrative literature review was to synthesize evidence that describes processes and conceptual models for assessing research impacts, with a focus on policy and practice impacts of public health research.

## Methods

### Literature review search strategy

The review involved keyword searches of electronic databases including MEDLINE (general medicine), CINAHL (nursing and allied health), PsycINFO (psychology and related behavioural and social sciences), EBM Reviews, Cochrane Database of Systematic Reviews 2005 to May 2013, and Google Scholar. Review search terms included ‘research impact’ OR ‘policy and practice’ AND ‘intervention research’ AND ‘translational research’ AND ‘health promotion’ AND ‘public health’.

The review included theoretical and opinion pieces, case studies, descriptive studies, frameworks and systematic reviews describing processes, and conceptual models for assessing research impact.

The review was conducted in two phases in July/August 2013. In phase 1, abstracts were retrieved and assessed against the review criteria. For abstracts that met the review criteria in phase 1, full papers were retrieved and were assessed for inclusion in the final review. Studies included in the review met the following criteria: i) published in English from January 1990 to June 2013; ii) described processes, theories, or frameworks associated with the assessment of research impact; and iii) were theoretical and opinion pieces, case studies, descriptive studies, frameworks, or systematic reviews.

**Figure 1:**
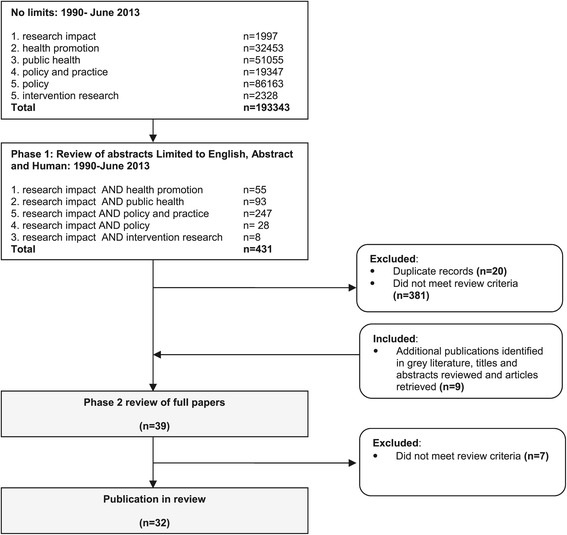
**Literature search process and numbers of papers identified, excluded, and included in the review of research impact assessment.**

Due the dearth of public health and health promotion-specific research impact assessment, papers with a focus on clinical or health services research impact assessment were included. The reference lists of the final papers were checked to ensure inclusion of further relevant papers; where such articles were considered relevant, they were included in the review. The search process is shown in Figure 1.

## Results

### Findings of the literature review

An initial review of abstracts in electronic databases against the inclusion criteria yielded 431 abstracts and searches of reference lists and the grey literature identified a further 9 documents. Of the 434 abstracts and documents reviewed, 39 met the inclusion criteria and full papers were retrieved. Upon review of the full publications against the review criteria, a further 7 papers were excluded as they did not meet the review criteria, leaving 32 publications in the review [8,9,13,15-44]. A summary of characteristics of studies included in the review that have a focus on processes, theories, or frameworks associated with the assessment of research impact including reference details, study type, domains of impact, methods and indicators, frameworks applied or proposed, and key lessons learned is provided in Additional file 1: Table S1.

### Study characteristics

The review identified 31 primary studies and 1 systematic review that met the review criteria. Six of the studies were reports found in the grey literature. Interestingly, 88% of studies that met the review criteria were published since 2006. The studies in the review included assessments of the impacts of a wide range of health-related research, including basic and biomedical research, clinical trials, health service research, as well as public health research. Six studies [22,23,34,36,40,43] had an explicit focus on assessing impacts of health promotion or public health research and 1 had a specific focus on intervention research impact assessment [36].

The majority of studies were conducted in Australia, United Kingdom, and North America, noting that the review was limited to studies published in English. The unit of assessment varied greatly from researchers (research teams [22] to whole institutions [15]) to research disciplines (e.g., prevention research [23], cancer research [41], tobacco control research [43]) or type of grants, for example, from public funding bodies [17,24]. The most frequently applied research methods across studies in rank order were publication and citation analysis, interviews with principal investigators, peer assessment, case studies, and document analysis. The nature of frameworks and methods used to measure research impacts will now be examined in greater detail.

### Frameworks and methods for measuring research impacts

Indices of traditional research productivity such as number of papers, impact factors of journals, and citations figured prominently in studies in the literature review [18,23,41].

Across the majority of studies in this review, research impact was assessed using multiple dimensions and methodological approaches. A total of 16 different impact assessment models were identified, with the ‘payback model’ being the most frequently used conceptual framework [15,24,29,31,44]. Other frequently used models included health economics frameworks [19,21,37], variants of Research Program Logic Models [9,35,42], and the Research Impact Framework [8,30]. A number of recent frameworks, including the Health Services Research Impact Framework [20] and the Banzi Health Research Impact Framework [13,34,36], are hybrids of previous conceptual approaches and categorize impacts and benefits in many dimensions, trying to integrate them. Commonly applied frameworks identified in the review, including the Payback model, Research Impact Framework, health economics models, and the new hybrid Health Research Impact Framework, will now be examined in greater detail.

The payback model was developed by Buxton and Hanney [45] and takes into account resources, research processes, primary outputs, dissemination, secondary outputs and applications, and benefits or final outcomes provided by the research. Categories of outcome in the ‘payback’ framework include i) knowledge production (journal articles, books/book chapters, conference proceeding, reports); ii) use of research in the research system (acquisition of formal qualifications by members of the research team, career advancement, and use of project findings for methodology in subsequent research); iii) use of research project findings in health system policy/decision making (findings used in policy/decision making at any level of the health service such as geographic level and organisation level); iv) application of the research findings through changed behaviour (changes in behaviour observed or expected through the application of findings to research-informed policies at a geographical, organisation and population level); v) factors influencing the utilization of research (impact of research dissemination in terms of policy/decision making/behavioural change); and vi) health/health service/economic benefits (improved service delivery, cost savings, improved health, or increased equity).

The model is usually applied as a semi-structured interview guide for researchers to identify the impact of their research and is often accompanied by bibliometric analysis and verification processes. The payback categories have been found to be applicable to assessing impact of research [15,24,29], especially the more proximal impacts on knowledge production, research targeting, capacity building and absorption, and informing practice, policy, and product development. The model has been found to be less effective in eliciting information about the longer term categories of impact on health and health sector benefits and economics [29].

The Research Impact Framework was developed in the UK by Kuruvilla et al. [8,30], and draws upon both the research impact literature and UK research assessment criteria for publically funded research, and was validated through empirical analysis of research projects at the London School of Hygiene & Tropical Medicine. The framework is built around four categories of impact, namely i) research related, ii) policy, iii) service, and iv) societal. Within each of these areas, further descriptive categories are identified. For example, the nature of research impact on policy can be described using the Weiss categorisation of ‘instrumental use’, where research findings drive policy-making; ‘mobilisation of support’, where research provides support for policy proposals; ‘conceptual use’, where research influences the concepts and language of policy deliberations; and ‘redefining/wider influence’, where research leads to rethinking and changing established practices and beliefs [30]. The framework is applied as a semi-structured interview guide for researchers to identify the impact of their research. Users of the framework have reported that it enables the systematic identification of a range of specific and verifiable impacts and allows consideration of the unintended effects of research [30].

The framework proposed by Banzi et al. [13] is an adaption of the Canadian Academy of Health Science impact model [25] in light of a systematic review and includes five broad categories of research impact, namely i) advancing knowledge, ii) capacity building, iii) informing decision-making, iv) health and other sector benefits, and v) broad socio-economic benefits. The Banzi framework proposes a set of indicators for each domain. To illustrate, indicators for informing decision making include citation in guidelines, policy documents, and plans; references used as background for successful funding proposals; consulting, support activity, and contributing to advisory committees; patents and industrial collaboration; packages of material and communication to key target audiences about findings. This multidimensional framework takes into account several aspects of research impact and use, as well as comprehensive analytical approaches including bibliometric analysis, surveys, audit, document review, case studies, and panel assessment. Panel assessments generally involve a process asking experts to assess the merits of research against impact criteria.

Economic models used to assess impacts of research varied from cost benefit analysis to return on investment and employed a variety of methods for determining economic benefits of research. The National Institutes of Medicine study in 1993 was among the first studies to attempt to systematically monetize the benefits of medical research. It provided estimates of savings for health care systems (direct costs) and savings for the community as a whole (indirect costs), and quantified benefits in terms of quality adjusted life years. On the other hand, the Deloitte Access Economics study [21] built on the foundations of the 1993 analysis to estimate the returns on investment in research in Australia for the main disease areas and employed of health system expenditure modelling and monetised total quality adjusted life years gained. According to Buxton et al. [19], measuring only health care savings is generally seen as too narrow a focus, and their analysis considered the benefits, or indirect cost savings, in avoiding lost production and the further activity stimulated by research.

The aforementioned models all attempted to quantify a mix of more proximal research and policy and practice impacts, as well as more distal societal and economic benefits of research. It is also interesting to note that across the studies in this review, only four [16,29,34,36] interviewed non-academic end-users of research in impact assessment processes, with the vast majority of studies relying on principal investigator interviews and/or peer review processes to assess impacts.

## Discussion

Comprehensive monitoring and measurement of research impact is a complex undertaking requiring the involvement of many actors within the research pipeline [13]. Interestingly, 90% of studies that met the review criteria were published since 2006, indicating that this is a new field of research. Given the dearth of literature on public health research impact assessment, this review included assessments of the impacts of a wide range of health-related research, including basic and biomedical research, clinical trials, and health service research as well as public health research.

The review of both the published and grey literature also revealed that there are a number of conceptual frameworks currently being applied that describe processes of assessing research impact. These frameworks differ in their terminology and approaches. The lack of a common understanding of terminology and metrics makes the task of quantifying research efforts, outputs, and, ultimately, performance in this area more difficult.

Most of the models identified in the review used multidimensional conceptualization and categorization of research impact. These multidimensional models, such as the Payback model, Research Impact Framework, and Banzi Health Research Impact Framework, shared common features including assessment of traditional research outputs, such as publication and research funding, but also a broader range of potential benefits, including capacity, building, policy and product development, and service development, as well as broader societal and economic impacts. Assessments that considered more than one category were valued for their ability to capture multifaceted impact processes [13,36,44]. Interestingly, these frameworks recognised that research often impacts not only in the country within which research is conducted, but also internationally. However, for practical reasons, most studies limited assessment and verification of impacts to a single country [19,34,36].

Several methods were used to practically assess research impact, including desk analysis, bibliometrics, panel assessments, interviews, and case studies. A number of studies highlighted the utility of case study methods noting that a considerable range of research paybacks and perspectives would not have been identified without employing a structured case study approach [13,36,44]. However, it was noted that case studies can be at risk of ‘conceptualization bias’ and ‘reporting bias’ especially when they are designed or carried out retrospectively [13]. The costs of conducting case studies can also be a barrier when assessing large volumes of research [13,36].

Despite recent efforts, little is known about the nature and mechanisms that underpin the influence that health research has on health policy or practice. This review suggests that, to date, most primary studies of health research impacts have been small scale case studies or reviews of medical and health services research funding [27,31,35,39,41], with only two studies offering comprehensive assessments of the policy and practice impacts of public health research, with both focusing on prevention research in Australia.

The first of these aforementioned studies examined impact of population health surveillance studies on obesity prevention policy and practice [34], while the second [36] examined the policy and practice impacts of intervention research funded through the NSW Health Promotion Demonstration Research Grants Scheme 2000–2006. Both of these studies utilised comprehensive mixed methods to assess impacts that included semi-structured interviews with both investigators and end-users, bibliometric analysis, document review, verification processes, and case studies. These studies concluded that research projects can achieve the greatest policy and practice impacts if they address proximal needs of the policy context by engaging end-users from the inception of research projects and utilizing existing policy networks and structures, as well as using a range of strategies to disseminate findings that go beyond traditional peer review publications.

This review suggests that the research sector often still uses bibliometric indices to assess research impacts, rather than measuring more enduring and arguably more important policy and practice outcomes [6]. However, governments are increasingly signaling that research metrics of research quality are insufficient to determine research value because they say little about real world benefits of research [10-12]. The Australian Excellence in Innovation trial [26] and the UK’s Research Excellence Framework trials [28,46] were commissioned by governments to determine the public benefit from research spending [10,16,47].

These attempts raise an important question of how to construct an impact assessment process that can assess multi-dimensional impacts while being feasible to implement on a system level. For example, can 28 indicators across 4 domains of Research Impact Framework be realistically measured in practice? This could also be said of the Research Impact Model [13], which has 26 indicators, and the Research Excellent Framework by Ovseiko et al. [38], which has a total of 20 impact indicators. If such methods are to be widely used in practice by research funders and academic institutions to assess research impacts, the right balance between comprehensiveness and feasibility must be struck.

Though a number of studies suggest it is difficult to determine longer-term societal and economic benefits of research as part of multi-dimensional research impact assessment processes [13,36,44], the health economic impact models presented in this review and the broader literature demonstrate that it is feasible to undertake these analyses, particularly if the right methods are used [19,21,37,48].

The review revealed that, where broader policy and practice impacts of research have been assessed in the literature, the vast majority of studies have relied on principal investigator interviews and/or peer review to assess impacts, instead of interviewing policymakers and other important end-users of research. This would seem to be a methodological weakness of previous research, as solely relying on principal investigator assessments, particularly of impacts of their own research, has an inherent bias, leaving the research impact assessment process open to ‘gilding the lily’. In light of this, future impact assessment processes should routinely engage end-users of research in interviews and assessment processes, but also include independent documentary verification, thus addressing methodological limitations of previous research.

One of the greatest practical issues in measuring research impact, including the impact of public health research, are the long lag times before impacts manifest. It has been observed that, on average, it takes over 6 years for research evidence to reach reviews, papers, and textbooks, and a further 9 years for this evidence to be implemented into practice [49]. In light of this, it is important to allow sufficient time for impacts to manifest, while not waiting so long that these impacts cannot be verified by stakeholders involved in the production and use of the research. Studies in this review have addressed this issue by only assessing studies that had been completed for at least 24 months [36].

As identified in previous research [13], a major challenge is attribution of impacts and understanding what would have happened without individual research activity or what some describe as the ‘counterfactual’. Creating a control situation for this type of research is difficult, but, where possible, identification of baseline measures and contextual factors is important in understanding what counterfactual situations may have arisen. Confidence in attribution of effects can be improved by undertaking independent verification of processes and engaging end-users in assessments instead of solely relying on investigators accounts of impacts [36].

The research described in this review has some limitations that merit closer examination. Given the paucity of research in this area, review criteria had to be adjusted to include assessment of impacts beyond public health research to include all health research. It was also challenging to make direct comparisons across studies mostly due to the heterogeneity of studies and the lack of a standard terminology, hence the broad definition of ‘research impact’ finally applied in the review criteria. Although the majority of studies were found in the traditional biomedical databases (i.e., Medline, etc.), 18% were found in the grey literature highlighting the importance of using multiple data sources in future review processes. Another methodological limitation also identified in previous reviews [13], is that we did not estimate the level of publication bias and selective publication in this emerging field. Finally, as our analysis included studies published up to June 2013, we may not have captured more recent approaches to impact assessment.

## Conclusions

Research impact assessment is a new field of scientific endeavour and typically impacts are assessed using mixed methodologies, including publication and citation analysis, interviews with principal investigators, peer assessment, case studies, and document analysis. The literature is characterised by an over reliance on bibliometric methods to assess research impact. Future impact assessment processes could be strengthened by routinely engaging the end-users of research in interviews and assessment processes. If multidimensional research impact assessment methods are to be widely used in practice by research funders and academic institutions, the right balance between comprehensiveness and feasibility must be determined.

## Additional file

Additional file 1: Table S1. Characteristics of studies focusing on processes, theories, or frameworks assessing research impact.
