# Supplementary material for: A narrative review of research impact assessment models and methods
Source: Health Res Policy Syst. 2015 Mar 18;13:18. doi: 10.1186/s12961-015-0003-1 (PMC4377031; doi:10.1186/s12961-015-0003-1)
Supplement: Additional file 1: Table S1. — Characteristics of studies focusing on processes, theories, or frameworks assessing research impact. [file 12961_2015_3_MOESM1_ESM.docx]

**Additional file 1: Table S1** Characteristics of studies focusing on processes, theories or frameworks assessing research impact.

| **Reference** | **Type of research assessed** | **Study type** | **Domains of impact** | **Methods and indicators** | **Frameworks applied or proposed** | **Key lessons learned** |
| --- | --- | --- | --- | --- | --- | --- |
| Aymerich et al (2012) [15] | Spanish network center for research in epidemiology and public health | Mixed methods research | Domains:   1. knowledge translation; 2. research capacity building; 3. potential healthcare benefit; 4. degree of objective fulfilment; 5. level of impact of research groups performance; and 6. wider social payback. | **Methods and indicators:**   - Self-reported assessment (Questionnaire to Investigators). - Peer review assessment (Questionnaire to external experts). | Payback Model | - The method showed a good discriminating ability that makes it possible to measure, reliably, the extent to which a project’s objectives were met as well as the degree to which the project contributed to enhance the group’s scientific performance and of its social payback. |
| Banzi et al (2011) [13] | Health research | Systematic review | Domains:   1. advancing knowledge; 2. capacity building; 3. informing decision-making; 4. health benefits; and 5. broad socio- economic benefits. | **Methods and indicators:**   - Bibliometric & citation analysis. - Desk analysis. - Database review. - Interviews. - Case studies. - Audit. - Assessment panels. - Econometrics. | Banzi Research Impact Model | - Evaluation of the research impact is a heterogeneous, and evolving discipline. - Multidimensional conceptual frameworks take into account several aspects of impact and use a mix of analytical approaches to measure impacts. - It remains to be clarified how different impact dimensions should be valued and balanced by assessors to fit them to their specific purposes and contexts.. |
| Barber et al (2012) [16] | Public involvement (community engagement) in research | Mixed methods research | Public involvement in research | **Methods:**   - Two-round Delphi study with pre-specified 80% consensus criterion. - Follow-up interviews with UK and international panellists from different settings, including universities, health and social care institutions and charitable organizations. | - | - It is feasible to evaluate the impact of public involvement on some research processes, outcomes and on key stakeholders. |
| Barker (2007) [17] | Research in the United Kingdom (UK) | Commentary | UK academic institutions  Three quality profiles are defined (panel decides the weight given to each profile):   1. research output (minimum 50%); 2. research context (minimum 5%); and 3. other indicators (minimum 5%). | **Indicators:**   - RAE1: staff information (volume and type of contracts, external collaborators), analysis of funding for research fellows; - RAE2: research output (publications, patents, reports, database, software); - RAE3: research scholarships - RAE4: attractiveness for external funding; and - RAE5a: information on groups of research (networking and research culture. | Research Assessment Exercise (RAE) 2008 | - RAE represents one of the most institutionalized forms of research evaluation in the OECD economies. It has become a primary means of concentrating resources for research in a relatively small number of universities. |
| Boyack & Jordan (2011) [18] | National Institutes of Health (NIH) grants | Citation analysis | Research impact using citation analysis | **Methods:**   - Articles linked to using the grant acknowledgment strings in PubMed using a parsing and matching process, with additional data from PubMed and citation counts from Scopus added to the linkage data.   **Indicators:**   - No of unique Articles. - No of multi-institute articles. - Percentage multi-institute articles. - Mean duration (years). - Percentage of grants with articles. - No of Scopus Articles   Average cites. | - | - The median time lag to publication for a new grant is 3 years. - Each grant contributes to approximately 1.7 articles per year, averaged over all grant types. |
| Buxton et al (2008) [19] | Medical Research in the United Kingdom (UK) (cardiovascular health and mental health) | Economic evaluation | 1. Health gains net of the health care costs of delivering them 2. Gross domestic product (GDP) gains (national income that results directly and indirectly from the medical research and the further activity stimulated by it). | **Methods:**   - Review of the economic evaluation literature. - Monetised the total Quality Adjusted Life Years (QALYs) gained by multiplying these estimates by published figures on the opportunity cost of a QALY within the current National Health Service budget - Estimates of the incremental health care costs associated with each intervention (multiplied by the numbers of users to quantify the incremental health care costs of each intervention). - Estimated ‘spillovers’ from public/charitable research between organisations and between sectors to estimate the impact of this research on the UK’s GDP.   **Indicators:**   - Total value of the QALYs gained from the specific interventions. - Proportion of UK health care benefit attributable to UK research. - Mean lag between research and impact. - Internal rates of return (IRRs)   on research investment from the value of the QALYs gained.   - GDP gains that result from increased public/charitable medical research. | Return on investment | - Public and private research leads to improved productivity and performance in the economy generally - The use of a standardised (and mapped) way of classifying research funding by funding agencies would aide future research impact assessment. - Further research needs to be undertaken to   understand the time lag between research expenditure and health gain.   - Deeper understanding of the international flows of knowledge and influence would be valuable. |
| Buykx et al (2012) [20] | Health Services Research | Literature review and critical appraisal of existing  models of research impact. | Elements:   1. research-related; 2. impact (advancing knowledge); 3. policy impact (informing decision making); 4. service impact (Improving health and health systems); and 5. societal impact (creating broad social and economic benefit). | **Methods and indicators:**   - Peer-reviewed articles (journal impact factor). - All other publications, clinical guidelines, recommendations,   policy briefs and so on.   - Presentations to academic conferences, decision makers, non-governmental organizations, consumer groups & public. - Media (e.g. press releases, Tweets, blogs). - Data sharing. - Subsequent grants received (type and quantity). - Follow on research by self or others. - PhD and postdoctoral completions. - Development, delivery and evaluation of capacity development program. - Active participation in policy networks (e.g. advocacy groups, think tanks, government advisors). - Validated measures to improve health care input and effectiveness. | Health Services Research Impact Framework | - Demonstrating accountability through recording research impact should be balanced with realistic expectations about the degree of certainty with which impact can be ‘proven’. - Framework yet to be tested but authors propose testing the utility of the framework by recording and monitoring the impact of their own research. |
| Deloitte Access Economics (2012) [21] | National Health and Medical Research Council (NHMRC) Research in Australia | Economic evaluation | Economic benefits:   - Health system expenditure savings - Return on investment | **Methods:**   - Health system expenditure modelling based on projected health care costs and research and development (R&D_ expenditure to derive return on investment. - Sensitivity analysis   **Indicators:**   - Health system expenditure saving - Net present value (NPV) of extra NHMRC expenditure ($bn) - Cents returned on the dollar | Return on investment | - It is feasible to conduct an extrapolated cost benefit analysis of medical research. - For every dollar spent on additional NHMRC R&D, seven cents would be returned in health expenditure savings in the future. |
| Derrick et al (2011) [22] | Australian Researchers  in Six Fields of Public Health | Mixed methods (citation analysis & peer assessment) | Research Impact and Influence | - Bibliometric indicators: h-index, m-index, m-quotient and q2-index. - Peer assessment of researcher influence. | - | - For 4 of the six fields the results showed a modest positive correlation between different research metrics and peer assessments of research influence. - Peer understanding of research influence within these fields differed from visibility in the mainstream, peer-reviewed scientific literature |
| Franks et al (2006)[23] | Prevention research | Citation analysis | Research impact | **Methods:**   - Research Centres asked to list up to15 articles considered the most important to have been published by its group in peer-reviewed journals between 1994 and 2004.   Each was also asked to name the journals it considered to be the most influential in its field(s) of interest.  **Indicators:**   - Impact factor. - Citations. - Impact half-life. | - | - Conventional bibliometric analysis to assess the scientific impact of public health prevention research is feasible, but of limited utility. - Assessment of impact on public health practice, policy, or on the health of populations, will require more than a bibliometric approach. |
| Graham et al (2012) [24] | Health research: Alberta Heritage Foundation for Medical Research | Mixed methods design | - Advancing - Knowledge - Building Capacity - Informing Decision- - Making; - Health; and - Broad Socio-Economic. | **Methods:**   - Citation analysis - Document review (quantitative and qualitative) mapped to the CAHS impact categories - Case studies   **Indicators:**  The impact categories are divided into a number of subcategories with 66 indicators mapped to each category or subcategory. | Canadian  Academy of Health Sciences (CAHS)  impact framework[25] (based on Payback Model) | - The model CAHS model can be applied at multiple levels from the micro (e.g. individual grantee projects, research programs, etc.), meso (e.g. organization) to the macro (e.g. contribution to the provincial innovation and health systems, etc.) and across different time intervals. - The framework is appropriate for evaluating impacts across the full spectrum of health research. |
| Group of Eight Australian (universities) and Australian Technology Network (2012)[26] | To measure the innovation dividend of research generated by Australian universities across areas of:   - Defence - Economic Development - Society (including health) - Environment | Mixed methods and commentary | Reach: The spread or breadth of influence or effect on the relevant constituencies  Significance: the intensity of the influence or effect | **Methods:**   - Citation analysis - Case studies - Panel assessment (made up of research and end users)   The Panels assessed each case study against *overall* Reach and Significance rather than assessing each separately.  Following assessment, a rating was assigned to each case study according to the following scale:   - A = Outstanding impacts - B = Very considerable impacts „. - C = Considerable impacts - D = Recognised but modest impacts - E = Of limited reach or significance. - Not classified – the impact was not underpinned by research or the link between the research and the claimed impact has not been demonstrated to the Panel’s satisfaction. | Excellence in Innovation | - It is possible to assess research impact across a broad range of disciplines. - Case study approaches can provide a compelling narrative of the impact of research. - Research impact could be assessed against an outcomes based system of classification. - Expert Panels comprising a majority of end-user stakeholders are able to assess research impact. - Panels should include an appropriate discipline mix covering the breadth of research impacts being considered. |
| Hanney et al (2007) [27] | NHS Health Technology Assessment  Programme | Mixed methods design | Research and policy and practice impacts | **Methods:**   - National Coordinating Centre for Health Technology Assessment (NCCHTA) documentation. - Surveys of principal investigators. - Detailed case studies. | - | - NCCHTA Program has had considerable impact in terms of knowledge generation and perceived impact on policy and to some extent on practice. - This high impact may have resulted partly from the NCCHTA Programme’s objectives, in that topics tend to be of relevance to the NHS and have policy customers. |
| Higher Education Funding Council for England (2011) [28] | High education funding in England | Mixed methods | Reach and significance of the impact or benefit | **Methods:**   - Case study submissions (include citations, documentary evidence) - Panel assessments | Research Excellence Framework  (REF) 2011 | - It is possible to   assess the impact of research through an approach based on expert review of case studies.   - UK funding bodies have decided that the REF will assess the ‘impact’ arising from research, alongside the ‘outputs’ and ‘environment’ elements - The framework provides recognition of economic and social benefits of excellent research |
| Kalucy et al (2009)[29] | Primary Care Research | Mixed methods. | - Advancing - Knowledge - Building Capacity - Informing Decision- - Making; - Health; and - Broad Socio-Economic. | **Methods:**   - Telephone interviews with research teams and nominated users of research, - bibliometric methods - documentary evidence of impact where possible | Payback Model | - It is feasible to use the Payback framework to determine the proximal impacts of primary health care research. - Though resource intensive, telephone interviews of chief investigators and nominated users provided rich information. |
| Kuruvilla et al (2006) [30] | Health research | Literature review and interviews with researchers | Framework domains:   1. research-related impacts; 2. policy impacts; 3. service impacts; 4. health and intersectoral; & 5. societal impacts. | **Methods:**   - Literature review. - Semi-structured interviews with principal investigators to develop narratives. - Case studies of research impact which were used to develop the framework.   **Indicators:**   - Type of problem/knowledge. - Research methods used. - Publications and papers. - Products, patents and translatability potential. - Research networks. - Leadership and awards. - Research management. - Communication. - Level of policy-making. - Type of policy. - Nature of policy impact. - Policy networks. - Political capital. - Type of services: health/ intersectoral. - Evidence-based practice. - Quality of care. - Information systems. - Services management. - Cost-containment & cost-effectiveness. - Knowledge, attitudes & behavior. - Health literacy. - Health status. - Equity and human rights. - Macroeconomic/related to the economy. - Social capital & empowerment. - Culture & art. - Sustainable outcomes. | Research Impact Framework | - The framework provides prompts and descriptive categories that can help researchers to systematically identify a range of specific and verifiable impacts related to their work (compared to ad hoc approaches they had previously used). - The standardized structure of the framework facilitates   comparison of research impacts across projects and time, which is useful from analytical, management and assessment perspectives. |
| Kuruvilla et al (2006) [8] | Health services research | Impact narrative (mixed methods) | As above | **Methods:**   - impact narrative of research projects involving: - semi-structured interviews with principal investigators - documentary analysis of the projects.   **Indicators:**  As above | Research Impact Framework | - Researchers were relatively easily and methodically able to identify and present impacts of their work. - Researchers’ narratives contained verifiable evidence and highlighted a wide range of areas in which health services and policy research has impact. - Factors thought to positively influence the impact of research included researchers’ involvement in research and policy networks, established track records in the field, and the ability to identify and use key influencing events, such as ‘policy windows’. |
| Kwan et al (2007) [31] | Hong Kong Health and Health Services Research Fund (HHSRF) | Mixed methods | Payback domains:   1. knowledge production; 2. research utilization; 3. informing policy and decision making; 4. application of the findings through changed behavior; and 5. health and health service benefit. | **Methods:**   - Surveys with principal investigators. - Bibliometric analysis.   **Indicators:**   - Publications per project. - Peer reviewed publications per project - Journal impact factor. - Journal ranking. - Citations per year. - Led to participation in health-related policy/advisory committees post research. - Completion. - Pre- and during- research process liaison with potential users. - Generated subsequent research. - Led to qualifications. - Led to career advancement - Findings used in policy making. - Findings expected to be used in policy making. - Led to changes in behavior. - Expected to lead to changes in behavior. - Reported health service benefit. - Expected future health service benefit. | Payback Model | - Multivariate analysis found participation of investigators in policy committees and liaison with potential users were significantly associated with reported health service benefit. - Payback outcomes were positively associated with the amount of funding awarded. - Further studies are needed to better understand the factors and pathways associated with the translation of research findings into practice. |
| Landry et al (2001) [32] | Research | Commentary | Ladder elements:   1. transmission (of research results to practitioners and policy makers); 2. cognition (reading and understanding); 3. reference (quoting of research results in reports, studies, actions); 4. effort (to adopt research results); 5. influence (on choices and decisions); and 6. Application. | - | Research  utilization  ladder | - The results suggest that there are barriers to climbing the research utilisation ladder and that these barriers are primarily located between the stage of no transmission and the stage of transmission. |
| Lavis et al (2003) [33] | Health research | Commentary | Model elements:   1. policy makers are the ones seeking research (user-pull); 2. researchers actively disseminating results (producer pull); and 3. researchers and policy-makers are both involved actively (exchange measures). | **Methods and Indicators:**   - Process measures (if limited   resources are available).   - Intermediate outcome measures (by performing surveys). - Outcome measures (by   performing cases studies). | Decision making  Impact model | - Research knowledge may be used in instrumental, conceptual or symbolic ways. - The proposed assessment tool as research can assist in better impact assessment and accountability in the health sector. |
| Laws et al (2013) [34] | Population health surveys | Mixed methods design | Domains:   1. advancing knowledge; 2. capacity building; 3. informing decision-making; 4. health and socioeconomic benefits | **Methods:**   - Semi-structured interviews with the chief investigators and - end-users - Bibliometric analysis and - Verification using documentary evidence. | Banzi Health Research Impact Model | - Factors influencing use of the findings: the perceived credibility of survey findings, dissemination strategies used; and contextual factors. - Highlighted the importance of engaging end-users from the inception of survey programs and utilizing existing policy networks and structures, and using a range of strategies to disseminate the findings that go beyond peer review publications. |
| Liebow et al (2009)[35] | National Institute of Environmental Health Sciences (NIEHS) Extramural Asthma  Research Program | Mixed methods design | Logic models elements:   1. Inputs; 2. Activities; and 3. Outputs and outcomes (Immediate, intermediate, ultimate). | **Methods:**   - A logic model tailored to inputs, outputs, and outcomes of the NIEHS asthma portfolio. - Data from existing National Institutes of Health (NIH) databases. - Bibliometric data. - Structured elicitation of expert judgment.   **Indicators:**   - Input indicators (NIH budget data, NIH asthma-related budget data, Non-NIH agency budget data). - Activity indicators (Grant awards, institutions, PIs). - Output indicators (Publications, Curricula, interventions, and outreach materials). - Outcome indicators (Citation database, Commissions, task forces, advisory panels, work groups, patents, drugs, legislation, Guidelines and care standards, Environmental changes related air quality, Asthma mortality, emergency department utilization, Hospitalization, Rescue medicine use, Quality of life indicators). | Research Impact Logic Model | - This logic model approach to research impact assessment demonstrates that it is possible to conceptualize program components, mine existing databases, and begin to show longer-term impacts of program results. - The next challenges will be to modify current data structures, improve the linkages among relevant databases, incorporate as much electronically available data as possible, and determine how to improve the quality and health impact of the science that we support. |
| Milat et al (2013)[36] | Health Promotion Applied Research | Mixed methods design | Domains:   1. advancing knowledge; 2. capacity building; 3. informing decision-making; 4. health and socioeconomic benefits | **Methods and indicators:**   - Bibliometric & citation analysis. - Desk analysis. - Database review. - Interviews with Investigators and End users of research - Case studies. - Assessment panel that engaged both research and end users.. | Banzi Health Research Impact Model | - Intervention research projects can achieve the greatest policy and practice impacts if they address proximal needs of the policy context by engaging end-users from the inception of projects and utilizing existing policy networks and structures, and using a range of strategies to disseminate findings that go beyond traditional peer review publications |
| National Institutes of Health (1993) [37] | Biomedical Research | Economic evaluation | Research impact evaluated in monetary  terms:   1. savings for health care systems (direct costs) 2. savings for the community on the whole (indirect costs) | **Indicators:**   - QALY - Profits | Cost benefit analysis | - Cost benefit analysis is a feasible method to assess research impact. |
| Ovseiko et al (2012) [38] | Academic clinical  medicine | Mixed methods design | Impact domains:   1. delivering highly skilled people; 2. creating new businesses, improving the performance of existing businesses, or commercializing new products or processes; 3. attracting R&D investment from global business; 4. better informed public policy-making or improved public services; 5. improved patient care or health outcomes; 6. progress towards sustainable development, including environmental sustainability; 7. cultural enrichment, including improved public engagement with science and research; 8. improved social welfare, social cohesion or national security; and 9. other quality of life benefits. | **Methods:**   - Existing administrative sources. - Online administrative survey carried out by the university’s Medical Sciences Division among n=289 clinical medicine faculty members.   **Indicators:**   - Staff movement between academia and industry. - Employment of post-doctoral researchers in industry. - Research contracts and income from industry. - Collaborative research with industry measured through   co-authored outputs.   - Income from intellectual property. - Success measures for spin-out companies. - Patents granted/licences awarded and brought to market. - Research income from overseas business. - Changes to legislation/ regulations/ government policy. - Participation on public policy advisory committees. - Influence on public policy debate. - Research income from the NHS and medical research charities. - Measures of improved health services. - Changes to clinical or healthcare training, practice or guidelines. - Development of new or improved drugs, treatments or other medical interventions; numbers of advanced phase clinical trials. - Changes to public behavior. - Measures of improved health outcomes. - Increased levels of public engagement with science and research. - Measures of improved social equity, inclusion or cohesion. - Application of new security technologies or practices. | Research Excellence  Framework | - The pilot exercise has confirmed that the majority of the proposed indicators have some validity, there are significant challenges in operationalizing and measuring these indicators reliably, as well as in comparing evidence of research impact across different cases in a standardized manner |
| Schapper et al (2012) [39] | Murdoch Childrens Research Institute, Australia | Mixed methods | 1. Knowledge creation; 2. Inputs to research; and 3. Commercial, clinical and public health outcomes. | **Methods:**   - Bibliometric analysis. - Document analysis. - Performance evaluation committee made up of peers.   **Indicators:**   - Publication activity – number of peer reviewed articles per evaluation year. - Publication impact – highly cited papers from the preceding 5-year period. - Technical papers that assist the translation of research into practice, e.g. policy, guidelines, books, book chapters. - Competitive peer-reviewed funding weighted by associated infrastructure received. - Research students trained. - Commercialization activity: contract funding gained through contracted research Commercialization activity: patents filed Research outcomes, adoption, implementation and evaluation. | Research Performance Evaluation | - The framework provides a fair and transparent means of disbursing internal funding. It is also a powerful tool for evaluating the Institute’s progress towards achieving its strategic goals, and is therefore a key driver for research excellence. |
| Spoth et al (2011) [40] | Family-Focused Prevention Science | Literature review and commentary | Translation Impact factors:   1. effectiveness; 2. extensiveness; 3. efficiency; and 4. engagement of public health impact. | **Indicators:**   - Analysis of long-term effects. - Effects across subgroups targeted by particular universal interventions. - Analysis of key mechanisms of effects. | PROSPER Model | - The model for community– university partnerships has the potential to facilitate the dissemination and public health impact of universal interventions to prevent underage drinking and other problem behaviors. - This model fits well within a comprehensive strategic framework for promoting effective prevention. |
| Sullivan et al (2011) [41] | United Kingdom cancer centres (UKCC) | Citation analysis | Research impacts | **Methods:**   - Publication and citation analysis.   **Indicators:**   - Research level (basic or clinical). - Potential and actual citation impact. - International collaborations. | - | - UK cancer centres focused on either basic cancer biology   or highly clinical trials received the highest aggregate citations. |
| Taylor & Bradbury-Jones (2011) [42] | Nursing research | Commentary | Impact mapping across:   1. Inputs; 2. Activities; 3. Outputs; 4. Outcomes; and 5. Impacts. | **Indicators:**   - Inputs (people, time and equipment). - Activities (actions that constitute the project). - Outputs are the direct results of the research (publications or dissemination to stakeholders). - Outcomes (behavior change or improved health outcomes). - Impacts (the outcomes less what would have happened anyway). | Research impact mapping | - The international principles of social impact assessment provide a meaningful framework in which to describe nursing research impact. |
| Warner & Tam (2012) [43] | Tobacco control research | Survey | Policy impact | **Methods:**   - Surveys of experts. - Case studies. | - | - Policy research goals established in 1992 have been largely realised. - For select tobacco control policies, research has made truly important contributions to saving lives. |
| Weiss (2007) [9] | Medical research | Commentary | Analyzes the ratio between input (resources), process (activity) and results  of research (products)  Domains of impact include:   1. initial benefits; 2. intermediate benefits; and 3. long-term benefits. | **Indicators:**:   - Publications. - Awareness of medical research. - Results in policy making. - Changes in practice. - Changes in well-being and health. | Weiss Logic Model | - Change from an output-based to an outcomes-based perspective is a critical step in examining the role that clinical scientists can play in decreasing the well documented efficacy- effectiveness gap. - Without this shift, there will be little incentive for researchers to look beyond the outputs of their work. |
| Wooding et al (2004) [44] | Arthritis research campaign (arc) funded research in the UK | Mixed methods | Payback domains:   1. Knowledge production 2. Research targeting and capacity building 3. Informing policy and product development 4. Health and health sector benefits 5. Wider economic benefit | **Methods:**   - Document review - Publication and citation analysis - Key informant interviews (Investigators and end users) - Case study analysis   **Indicators:**   - Number of publications - Citations - Proportion of citations internationally - Number of partnerships - Research funding - Research capacity building - Generated subsequent research. - Led to qualifications. - Led to career advancement - Findings used policy and product development. - Reported health service benefit - Economic benefits | Payback model | - There was a considerable range of research paybacks and these would not have been identified without employing structured case study approach. - When translation of research into developments of practical value to patients occurs it is largely due to the conviction, effort and personal networks of a particular investigator, and is not associated with the type or mode of the funding stream or the bibliometric impact of the investigator - payback framework could be operationalized and embedded. - There was as no quantifiable evidence of broader economic returns arising from the arc-sponsored research. |
